# What role do dauciform roots play? Responses of *Carex filispica* to trampling in alpine meadows based on functional traits

**DOI:** 10.1002/ece3.9875

**Published:** 2023-03-08

**Authors:** Rong Fan, Jinguo Hua, Yulin Huang, Jiayi Lin, Wenli Ji

**Affiliations:** ^1^ College of Landscape Architecture and Arts Northwest A&F University Yangling Shaanxi China

**Keywords:** alpine meadows, *Cyperaceae*, dauciform roots, functional traits, intraspecific variance, trampling

## Abstract

In China, dauciform roots were hardly studied and only reported in alpine meadows, where sedges showed a different tendency from other functional groups such as grasses and forbs with degradation. In addition, *Carex* species were proved to have shifting scaling relationships among LES (leaf economics spectrum) traits under disturbance. So, are these unique performances of sedges related to the presence of dauciform roots, and if so, how? An alpine meadow dominated by *Carex filispica* in Baima Snow Mountain was selected, and quantitative trampling was performed (0, 50, 200, and 500 passes). The cover and dauciform root properties of *Carex filispica* were measured, as well as the morphological, chemical traits and biomass of leaves and roots, their correlations and the differences between individuals with and without dauciform roots were analyzed. After the trampling, individuals with dauciform roots showed multiple resource‐acquisitive traits: Larger, thicker leaves, more aboveground biomass, higher efficiency of nutrient utilization, and slenderer roots. Additionally, they had a tighter correlation among belowground biomass, morphological and chemical traits, as well as dauciform root properties and morphology of leaves, suggesting that their traits were more related than those without dauciform roots. The presence of dauciform roots in *Carex filispica* was related to advantages in multiple traits after trampling, which is consistent with and might be responsible for the unique performances of sedges.

## Graphical abstract

This study contributes to our understanding of the impacts of dauciform roots (DRs) under natural disturbed environments. Individuals with DRs showed a significantly different performance, relating to advantages in multiple traits, which might be responsible for the unique performance of sedges found in alpine meadows.

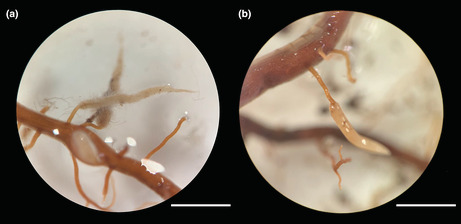

## 1 INTRODUCTION

To adapt to environmental diversity, all plants face the challenge to balance their competing needs with limited resources. As a result, diverse life strategies are shaped, which can be quantified by multiple functional trait dimensions (Reich, 2014). Functional traits of plants usually show a common pattern of co‐variation rather than varying independently, through which plants adjust their strategies of resource utilization and allocation to cope with resource limitation (Kühner & Kleyer, 2008; Watson & Szathmáry, 2016). In 2004, the leaf economics spectrum (LES) identified the functional integration among multiple leaf traits (Wright et al., 2004), which was later extended to a “global spectrum of plant form and function” involving leaf, stem, and root traits (Díaz et al., 2016). Yet, a recent study with 14 *Carex* species showed that the scaling relationship among LES traits expressed per unit area vs mass in these *Carex* species tended to shift with changes in leaf mass per area, which helped to sustain growth under resource (P and insolation) limitation (Ji et al., 2020), and this intraspecific variability of leaf traits was consistent with the costs of dauciform roots, a special root trait in *Carex* species (Gusewell & Schroth, 2017).

Dauciform roots (DRs) are a special root trait that is most known to be found in the *Cyperaceae* (Shane et al., 2005). DRs are short‐lived roots that release bound phosphorus (P) through root exudates (Gerke, 2015; Lambers et al., 2006), providing those sedges with an eco‐physiological advantage in P‐acquisition (Playsted et al., 2006; Shane et al., 2005). Supposedly, dauciform roots are formed under P deficiency (Gusewell, 2017), yet so far, species with DRs did not appear to occur at more P‐limited sites than those without them (Gusewell & Schroth, 2017), and the lack of research from comparative regions contributes to the unclearness of this strategy (Laliberté, 2017).

In China, DRs are poorly studied and only reported to be found in the alpine meadows in Yunnan Province (Gao & Yang, 2010), which are being increasingly degraded (Wang et al., 2020). As major human disturbance, overgrazing and tourism trampling had been proved to have dramatic effects on the degradation in small scales (Ballantyne et al., 2014; Dong et al., 2013; Henn et al., 2018; Liu, Mi, et al., 2018; Zhou et al., 2018). While causing direct damage to the aboveground parts of vegetation, the trampling also leads to instant changes in soil physical properties, resulting in a vicious cycle in which the growth of roots, the microenvironment and soil chemical properties all suffer (Beylich et al., 2010; Głąb, 2014). What is noteworthy is that, not only do *Cyperaceae* species appear to have a shifting scaling relationship among LES traits (Ji et al., 2020), but they are also found with a unique performance in these degrading alpine meadows: There are multiple studies in China, suggesting that along with the degradation of alpine meadows, the leaf area and aboveground biomass of grasses and forbs are always significantly reduced, while for sedges, those traits remain unchanged, vary slightly, or even show an opposite trend (Hao et al., 2020; Ma et al., 2010; Zhou et al., 2012). These findings invite the question: Are the unusual aboveground advantages of sedges related to DRs? We hypothesized that the unusual increase in leaf area and aboveground biomass of sedges with degradation is caused by the presence of DRs: Plants are having a hard time capturing resource in less ideal environments, while DRs in sedges contribute to their resource‐acquisitive advantages in the competing community and therefore their survival, so that the growth of sedges as a whole can be maintained or even improved.

To confirm this hypothesis, an alpine meadow dominated by *Carex filispica* in Baima Snow Mountain, Yunnan Province, was selected and quantitative trampling (0, 50, 200, and 500 passes) was performed to simulate the change of soil physical properties in small‐scale degradation. We analyzed the responding pattern of *Carex filispica* to trampling and compared the correlation among the functional traits to explore the role of DRs in this process, making an attempt to explain their unusual advantages in degradation and providing a future reference for further research on dauciform roots.

## 2 METHODS

### 2.1 Study area

The selected study site is in Baima Snow Mountain National Nature Reserve, which located in Deqin County of Yunnan Province with a cold temperate mountain monsoon climate; the annual mean temperature and precipitation are −1.0°C and 600‐650 mm, respectively (Gao & Yang, 2016). The climate there varies with altitude, leading to hot dry valleys, cold mountains, and a significantly vertically distributed vegetation. There is a pass located in the middle of reserve with National Highway 214 passing through. The pass area is mainly an alpine shrub‐meadow zone, which owing to the convenient location, and has a high intensity of human activities.

The site was selected at the pass (4320 m, 28°20′9″ N, 99°4′36″ E), about 300 m away from Highway 214, in an area with moderate disturbance of grazing and human trampling. The alpine meadow is dominated by *Carex filispica* and *Polygonum viviparum* and contains *Eleocharis yokoscensis*, *Gentiana scabra*, *Veronica didyma*, et al.

### 2.2 Experimental design and sampling

To compare the impacts of different intensities of trampling on *C. filispica*, a flat area with relatively well‐grown, even vegetation was selected and quantitative trampling was performed in September 2021. Six replicate blocks of 5 m long and 2 m wide were randomly laid out across the site, each block was at least 1 m from others to avoid cross‐interference, and each block was then subdivided into four transects (0.5 m wide, 2 m long, separated by 1 m gaps) for four treatments: control group with no trampling, 50, 200, and 500 passes (a one‐way trip along the transect is counted as one pass).

The six blocks were not set in a line, and the four treatments in each block were performed in the same order, lowest to highest trampling intensity. Three participants with similar weight of around 90 kg performed the treatment, two blocks each. Since there is no significant difference between trampling at one time and at multiple times (Bayfield, 1979), the trampling was performed in 1 day. After the treatments, the site was left surrounded by ropes with a sign, so there was no additional tourism trampling, but the free grazing situation could not be fully guaranteed.

### 2.3 Measurement

The absolute cover of vegetation community and *C. filispica* in each transect was measured before the trampling. The pictures of each transect were taken and imported into Photoshop 2020, where both the absolute cover of vegetation community and *C. filispica* were measured using a 5 × 20 grid, which proportionately covered each transect (0.5 m wide, 2 m long) with 100 intersections, each intersection of the grid with vegetation was recorded as a “hit,” and then multiplied by 100 to generate absolute cover values. The responses to damage had been stabilized after 2 weeks (Cole & Bayfield, 1993), when the absolute cover was measured again with the same method.

Two weeks after the trampling, morphological traits of *C. filispica* were measured: The thickness of leaves was measured with a vernier caliper, main veins included; the length, average width, and maximum width of leaves were measured by LI‐COR portable leaf area meter. The plants and surrounding soil plot of 10 × 10 × 10 cm each were excavated, which, owing to the shallow‐rooted situation, nearly contains the whole root system. All 378 individuals of *C. filispica* were then gently separated from the soil, washed, and sorted by whether they had DRs, after which 177 individuals with DRs were observed under a stereoscopic microscope, and the amount, density, size, color, and hair presence of DRs were recorded. The color of DRs was rated on a 5‐point scale, brightest to darkest (1 = White, 2 = Light yellow, 3 = Tawny, 4 = Dark brown, 5 = Black). The formula of DR density is as follows: total amount of DRs/ the dry weight of roots. Using a LA‐S root analyzer, we measured the total length, surface area, volume, and average diameter of the whole root system of 3 individuals with and without DRs per transect, respectively.

The plants were dried and the average biomass of the aboveground parts (leaves and fruits) and belowground parts (roots and rhizomes) were measured, respectively, after which they were ground up separately to measure the organic carbon (OC), total nitrogen (TN), and total phosphorus (TP) content. OC was determined by the potassium dichromate wet‐oxidation method, TN was determined by the Indophenol blue colorimetric method after digested with H_2_SO_4_‐H_2_O_2_, and TP was determined by the vanadium molybdate yellow colorimetric method after digested with H_2_SO_4_‐H_2_O_2_.

### 2.4 Statistical analyses

To exclude the influence of other factors such as season and weather, the absolute cover of *C. filispica* was converted to absolute cover difference (ACD) and relative cover difference (RCD). ACD reflects the difference in the absolute cover of *C. filispica* between different trampling intensities and the control group; RCD shows the difference in relative cover: A larger RCD suggests that *C. filispica* is less affected compared with the entire community. The calculation formulas are as follows:

$$\begin{array}{c} \text{Relative cover}=\text{Absolute cover of}\;\text{C. filispica}/\text{absolute cover of the community} \end{array}$$

$$\begin{array}{c} \mathrm{ACD}=\left(\text{absolute cover after trampling}/\text{absolute cover before trampling}\right)\\ \;\times \left(\text{absolute cover before trampling of control group}/\text{absolute cover after trampling of control group}\right)\times 100 \end{array}$$

$$\begin{array}{c} \mathrm{RCD}=\left(\text{relative cover after trampling}/\text{relative cover before trampling}\right)\\ \;\times \left(\text{relative cover before trampling of control group}/\text{relative cover after trampling of control group}\right)\times 100 \end{array}$$

All analyses were performed using SPSS Statistics (ver 19.0, IBM), and figures were made using Origin (ver 2022, OriginLab, USA). One‐way ANOVAs were used to analyze the effects of trampling on the coverage of *C. filispica*, biomass, DR properties, chemical traits, and root properties. The biomass and all root properties were subjected to a two‐way ANOVA with the intensities of trampling and DR presence (individuals with or without DRs). Statistical significances of the correlations among the chemical properties and root properties of *C. filispica* were tested by Pearson correlation, as well as the relationship among the properties of DRs and morphological traits.

## 3 RESULTS

### 3.1 Cover differences of *C. filispica*

Before the trampling, there was no significant difference in the total absolute cover of vegetation community or the absolute cover of *C. filispica* among the transects. Two weeks later, trampling with different intensities resulted in a significant difference in the total vegetation cover (*p* = .022) and showed a significant decrease after 500 passes, while the absolute cover of *C. filispica* showed no significant change (*p* = .494), as well as the ACD and RCD of *C. filispica* (Table 1).

**TABLE 1:** Comparison of total absolute cover of vegetation community, absolute cover, absolute cover difference (ACD), and relative cover difference (RCD) values of *C. filispica* under different trampling intensities.

	Trampling intensity				*F*	*p*
	0	50	200	500		
Total Absolute Cover (%)	78.33 ± 1.31a	82.50 ± 3.17a	82.17 ± 2.50a	67.83 ± 5.37b	4.008	.022*
C *C. filispica* Absolute cover (%)	31.83 ± 4.08a	38.17 ± 3.38a	37.50 ± 2.11a	32.67 ± 4.31a	0.829	.494
*C. filispica* ACD (%)	100.00 ± 0.00a	101.37 ± 12.19a	88.99 ± 6.57a	79.90 ± 10.92a	1.312	.298
*C. filispica* RCD (%)	100.00 ± 0.00a	98.58 ± 10.49a	89.78 ± 7.30a	90.61 ± 10.23a	0.419	.742

*Note*: Values represent the means of replicates ± SE (standard errors). Different letters indicate a significant difference among trampling intensities (*p* < .05). *F*, degrees of freedom; *p*, *p* values.

* Indicates a significant difference (*p* < .05).

### 3.2 Morphological traits of DRs and leaves

**FIGURE 1:**
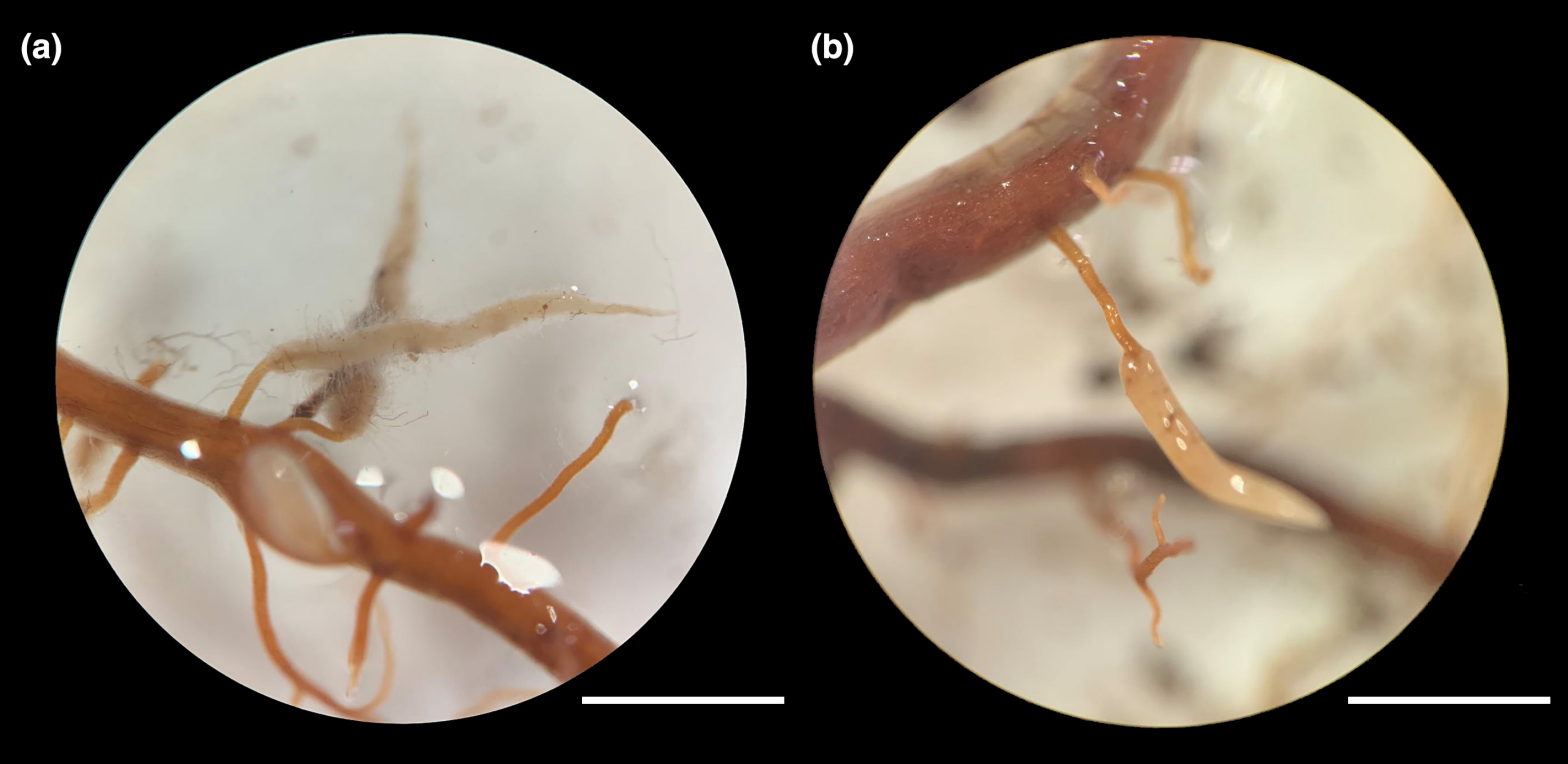
Morphology of dauciform roots from *C. filispica*. (a) Dauciform roots with hairs. (b) Dauciform roots without hairs. Bars = 1 mm.

**FIGURE 2:**
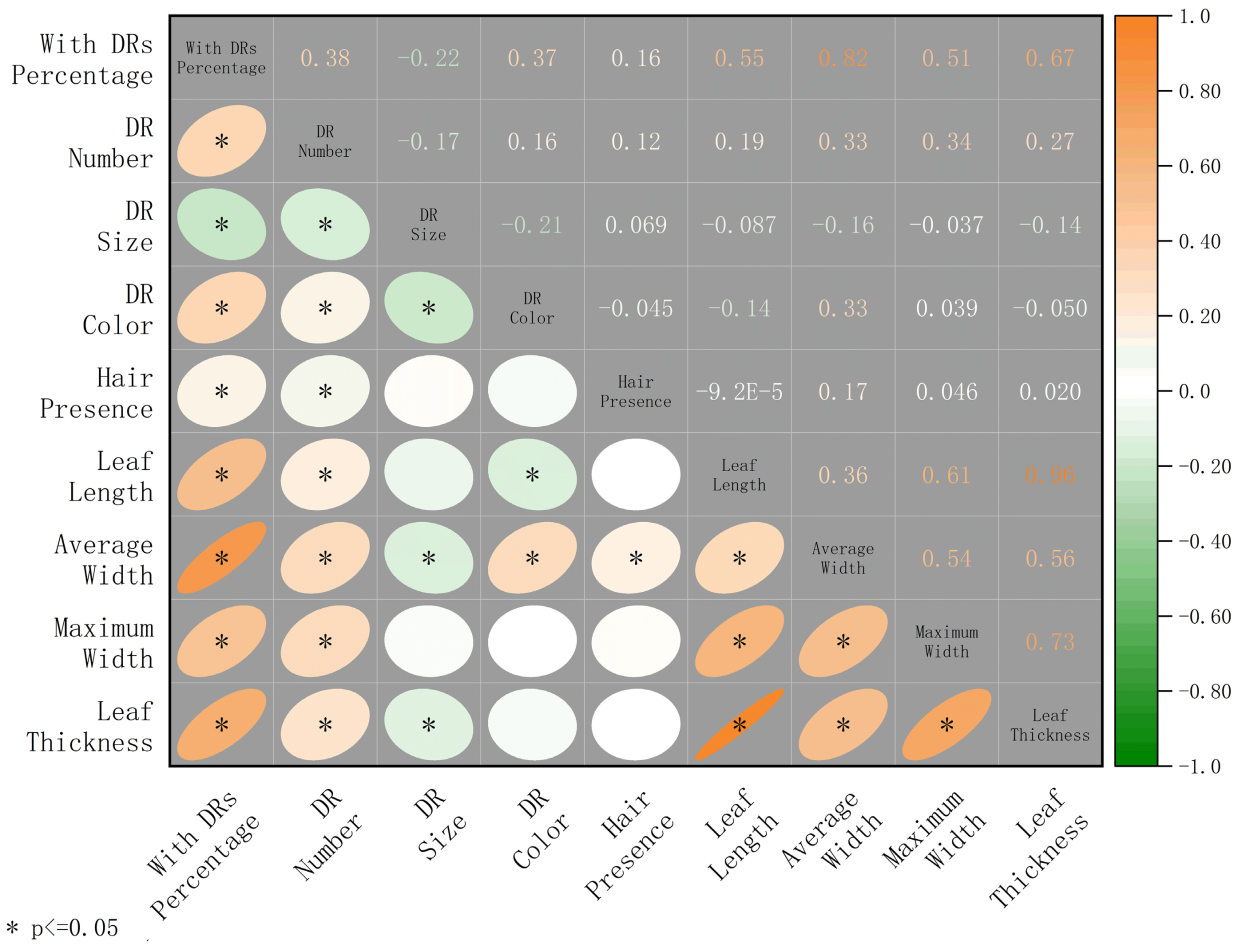
Relationships among morphological traits of dauciform roots (DRs) and leaves, described by Pearson correlation coefficients. * indicates a significant correlation (*p* < .05).

Both DRs with and without hairs were found in our study (Figure 1). Significant differences in DRs appeared to be driven by changes in trampling intensity. The percentage of individuals with DRs had a significant difference between 0 and 50 passes, which showed an increase after trampling, same as hair presence, the number and density of DRs (Table 2). This result was also consistent with Figure 2: The number of DRs and hair presence showed a positive correlation with the percentage of individuals with DRs. In addition, the size and color of DRs showed significant differences as well: DR size was larger after 200 passes, and the color showed a downward trend (Table 2).

**TABLE 2:** Comparison of dauciform root (DR) properties values under different trampling intensities.

	Trampling intensity				*F*	*p*
	0	50	200	500		
With DRs Percentage (%)	25.36 ± 4.95b	68.19 ± 4.36a	38.91. ± 9.09ab	49.36 ± 15.55ab	3.546	.126
DR Number (numbers per plant)	4.27 ± 0.83b	6.93 ± 0.54a	3.93 ± 0.67b	4.91 ± 0.47b	4.845	.003*
DR Density (numbers g^−1^ DW)	6.33 ± 3.07b	36.15 ± 10.03a	9.43 ± 5.99ab	17.85 ± 7.85ab	3.455	.131
DR Size (mm)	1.89 ± 0.14bc	1.53 ± 0.10c	3.01 ± 0.26a	2.24 ± 0.17b	14.425	.000*
DR Color (point)	3.37 ± 0.22ab	3.77 ± 0.13a	2.75 ± 0.12c	3.33 ± 0.12b	8.921	.000*
Hair presence (%)	80.00 ± 7.43b	93.64 ± 2.34a	88.24 ± 4.56ab	93.83 ± 2.69a	2.270	.081

*Note*: Values represent the means of replicates ± SE (standard errors). Different letters indicate a significant difference among trampling intensities (*p* < .05). *F*, degrees of freedom; *p*, *p* values.

* Indicates a significant difference (*p* < .05).

The morphological traits of leaves showed a significantly positive correlation between the proportion of individuals with DRs and the number of DRs (Figure 2). To be specific, the leaf length, average width, maximum width, and thickness all increased with the amount of DRs.

### 3.3 Aboveground and belowground biomass

The average biomass of all *C. filispica* individuals showed a negative correlation with trampling intensity (*r* = −.725, *p* = .042), suggesting that plants tended to have smaller size and lower biomass as the trampling intensified. Two‐way ANOVA showed that DR presence had a significant effect on the average aboveground biomass (*p* = .020), as did trampling intensity (*p* = .002), and the interaction of DR × trampling was also significant (*p* = .010), that is, the responses of aboveground biomass to trampling differed significantly between individuals with and without DRs (Table 3).

**TABLE 3:** Two‐way analysis of variance result for the effects of dauciform root (DR) presence, trampling intensity, and their interaction on the aboveground, belowground, and total biomass.

	Aboveground biomass		Belowground biomass		Total biomass	
	*F*	*p*	*F*	*p*	*F*	*p*
DR presence	8.297	.020*	0.494	.502	1.649	.235
Trampling intensity	13.076	.002*	0.943	.464	2.740	.113
DR× trampling intensity	7.484	.010*	0.476	.707	1.008	.438

Abbreviations: F degrees of freedom; *p*, *p* values.

* Indicates a significant difference (*p* < .05).

**FIGURE 3:**
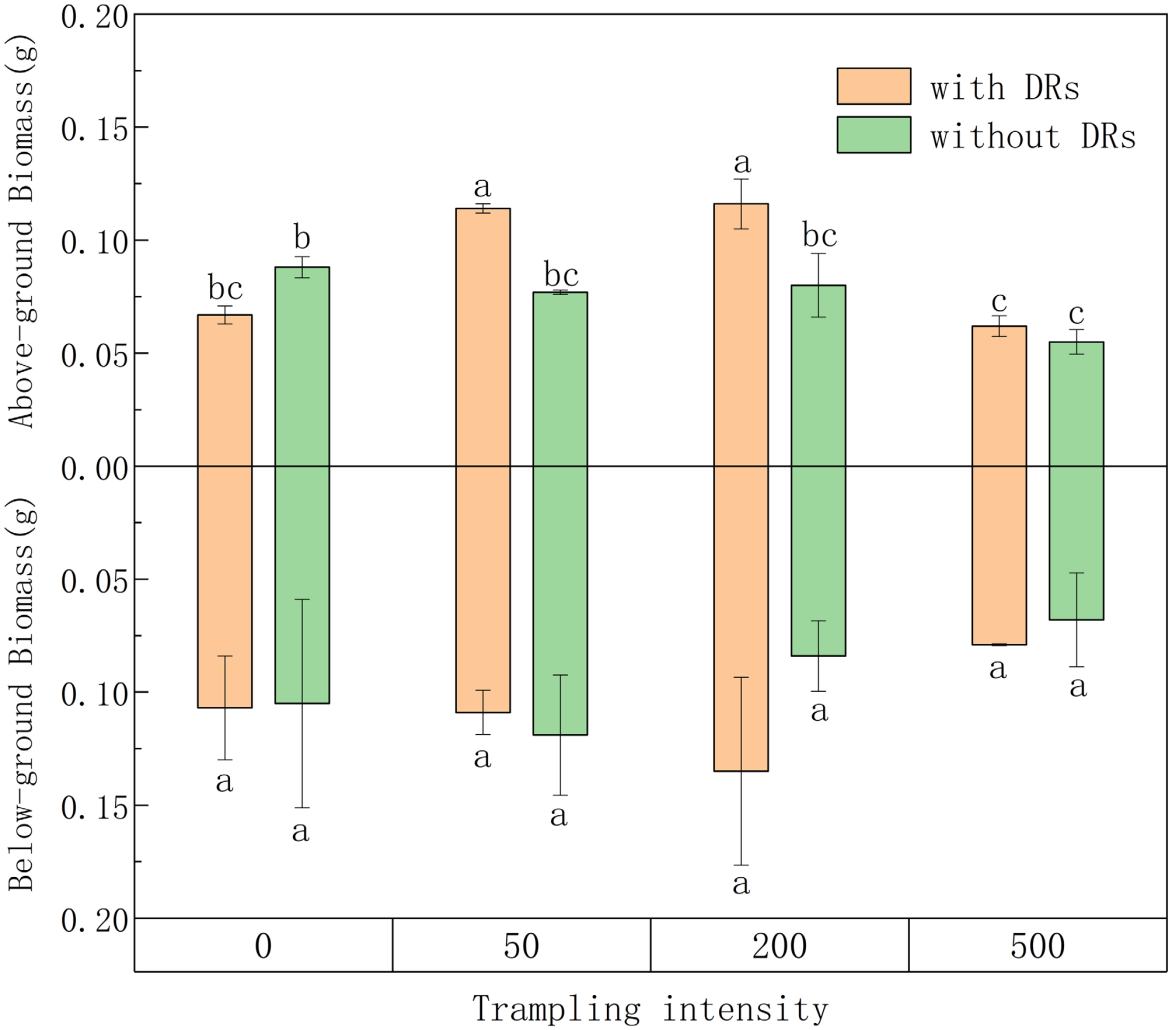
Differences between individuals with and without dauciform roots (DRs) under different trampling intensities in relation to average aboveground and belowground biomass per plant. Bars represent means ± SE (standard errors). Different letters indicate a significant difference among trampling intensities (*p* < .05).

As can be seen in Figure 3, with no extra disturbance, the aboveground biomass and belowground biomass of individuals with and without DRs did not differ. After 50 and 200 passes, individuals with DRs had significantly larger aboveground biomass, compared with control group and individuals without DRs under same disturbance, while belowground biomass remained steady. After 500 passes, the aboveground biomass of individuals without DRs had suffered and showed a significant difference from the control group, while those with DRs showed no such difference.

### 3.4 Chemical traits

**FIGURE 4:**
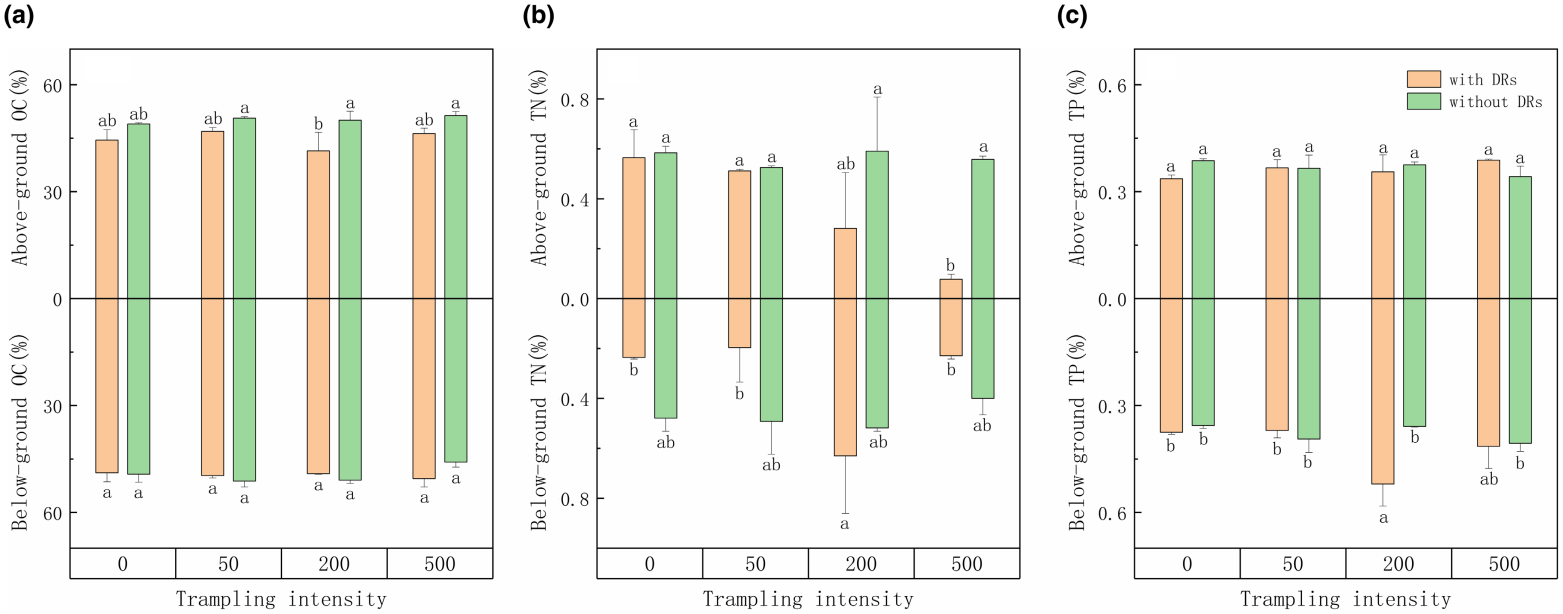
Differences among different trampling intensities in relation to (a) below/aboveground organic carbon (OC) content; (b) below/aboveground total nitrogen (TN) content; (c) below/aboveground total phosphorus (TP) content. Bars represent means±SE. Different letters indicate a significant difference among trampling intensities (*p* < .05).

**FIGURE 5:**
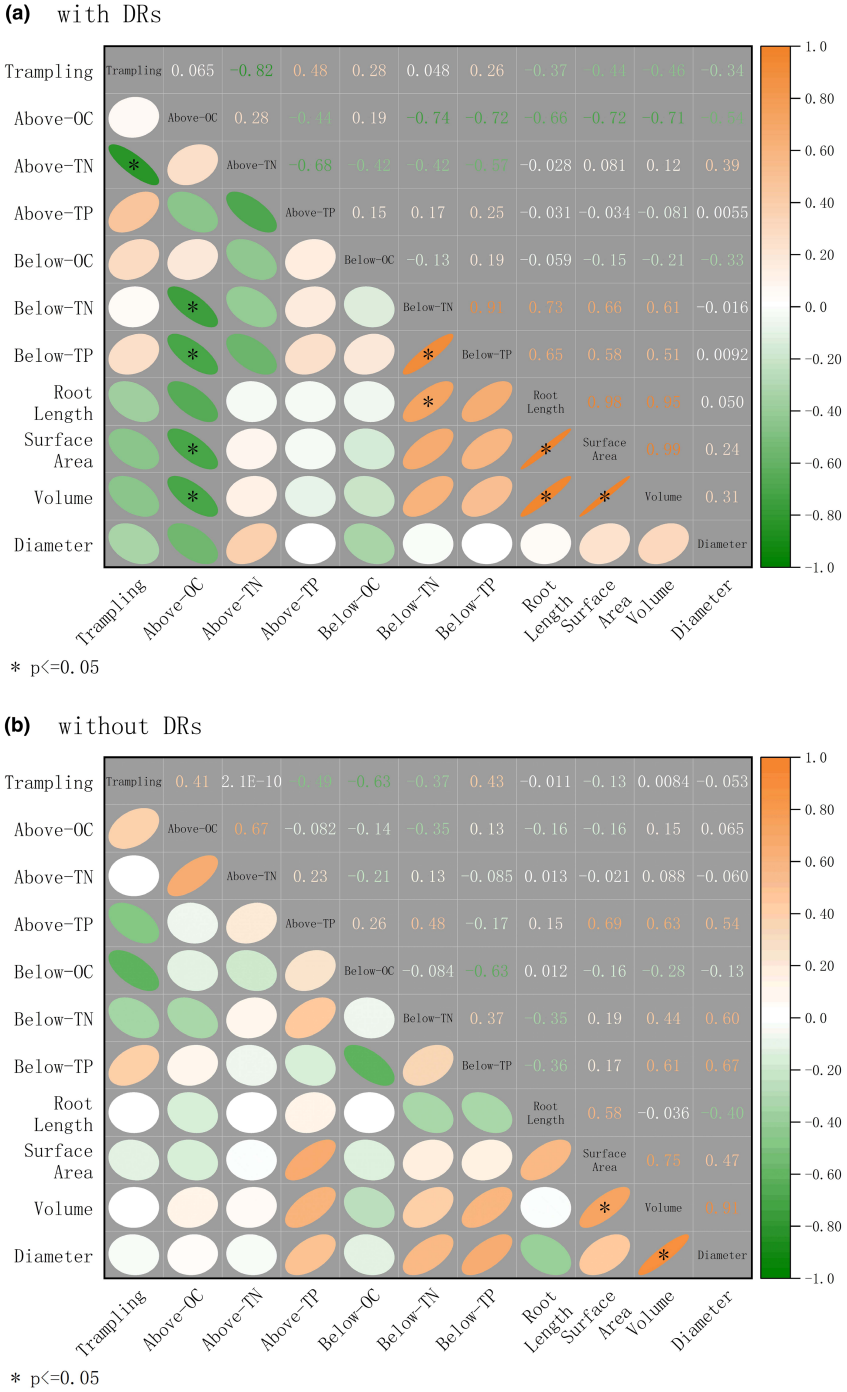
Relationships among trampling intensity, chemical traits, and root properties of *C. filispica*, described by Pearson correlation coefficients, separated by dauciform root (DR) presence. (a) relationships of individuals with DRs. (b) relationships of individuals without DRs. OC, organic carbon; TN, total nitrogen; TP, total phosphorus. * indicates a significant correlation (*p* < .05).

As trampling intensified, the aboveground TN content of individuals with DRs had a sharp decrease and showed a significantly negative correlation with trampling intensity, while individuals without DRs stayed stable and showed no such correlation (Figures 4b and 5). Contrasting with the dramatic decrease in TN, the aboveground OC and TP content of individuals with DRs stayed steady (Figure 4). Less N, stable OC and stable P led to a dramatic downward trend in the N:P ratios of aboveground of individuals with DRs; meanwhile, their C:N ratios increased significantly and were considerably different from individuals without DRs which stayed stable.

As for the belowground parts, the TN of individuals with DRs showed a significant difference after 200 passes and was significantly higher than other treatments (Figure 4b), which was also the fact for belowground TP (Figure 4c). Interestingly, these trends were also consistent with the root length of individuals with DRs: a relationship that did not exist in individuals without DRs (Figure 5).

### 3.5 Root properties

There was no significant effect of DR presence or trampling intensity on the measured root properties, nor did the interaction of DR × trampling intensity, that is, in total, the responses of root properties to trampling intensity did not differ between individuals with and without DRs (Table 4).

**TABLE 4:** Two‐way analysis of variance result for the effects of dauciform root (DR) presence, trampling intensity, and their interaction on the total root length, surface area, volume, and average diameter.

	Length		Surface area		Volume		Average diameter	
	*F*	*p*	*F*	*p*	*F*	*p*	*F*	*p*
DR presence	2.551	.118	0.094	.761	0.479	.493	2.934	.094
Trampling intensity	2.067	.120	1.136	.346	0.136	.938	0.841	.480
DR× trampling intensity	2.131	.111	1.584	.208	1.231	.311	1.510	.227

Abbreviations: *F*, degrees of freedom; *p*, *p* values.

**FIGURE 6:**
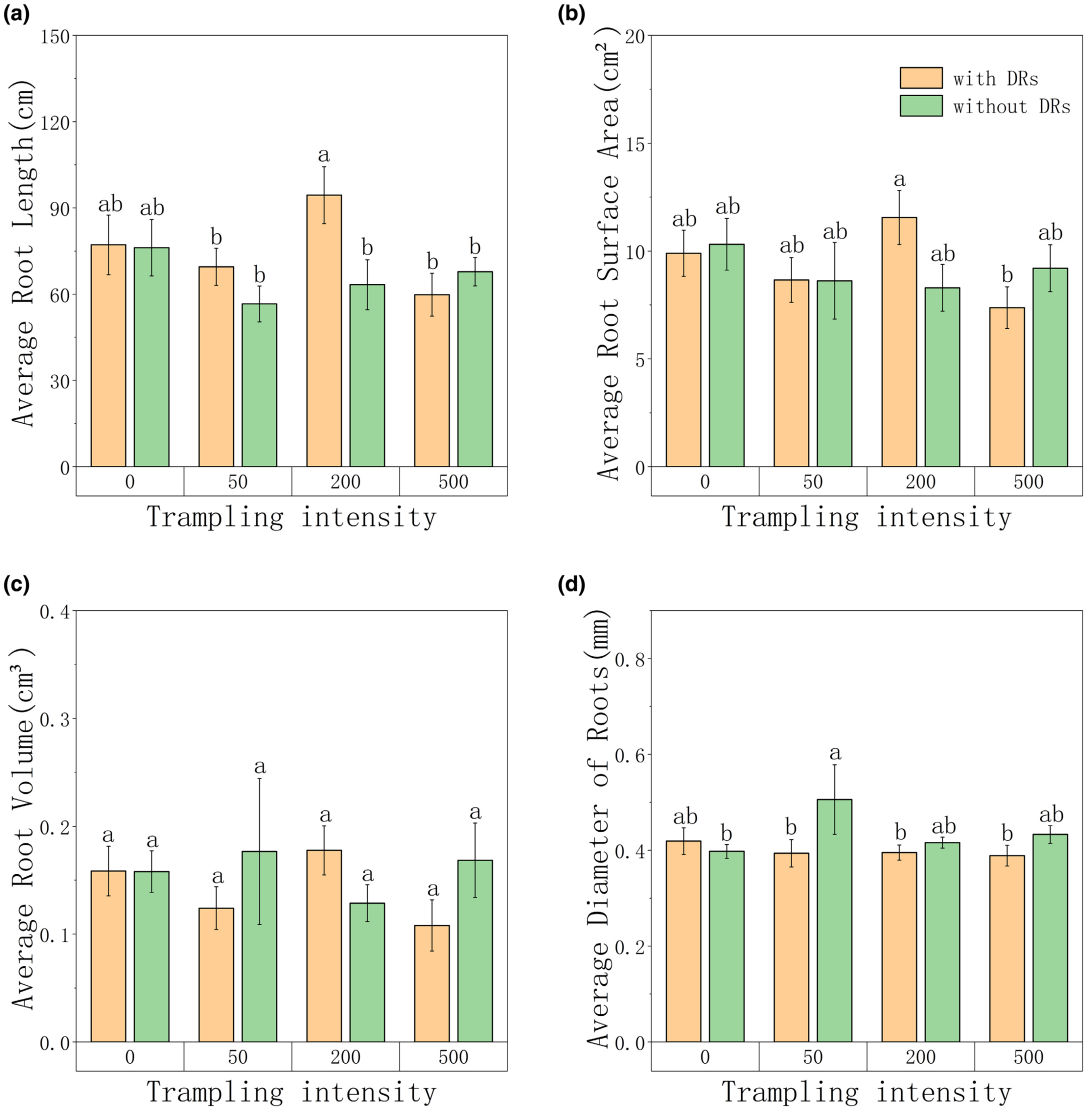
Differences among different trampling intensities in relation to (a) average root length; (b) average root surface area; (c) average root volume; (d) average diameter of roots. Bars represent means±SE. Different letters indicate a significant difference among trampling intensities (*p* < .05).

As shown in Figure 6, after 200 passes, the values of root length and surface area of individuals with DRs were significantly larger than other treatments, and the root volume showed no significant difference. In total, individuals with DRs had a higher root length, a larger surface area, and a lower average diameter (Figure 6), which were all the characteristics of slender roots. What is worth noting is that the length, surface area, and volume of roots of individuals with DRs showed an identical trend with belowground biomass, TN and TP content, which reached the peak after 200 passes and declined after 500 passes (Figures 3, 4 and 6), showing a positive correlation (Figure 5) and suggesting a same changing pattern of belowground parts.

## 4 DISCUSSION

### 4.1 High adaptability with an increasing DR proportion

Alpine plant communities were found to be less resistant to trampling, compared with lower altitudes (Ballantyne et al., 2014). Trampling often causes a decrease in aboveground biomass and cover (Hill & Pickering, 2009; Pescott & Stewart, 2014; Xu et al., 2014), as well as a transfer of competitiveness from sensitive species to more tolerant ones (Guo et al., 2019; Miehe et al., 2019). In our study, as the trampling intensified, the absolute cover of the vegetation community significantly suffered, while the absolute cover, the ACD and RCD of *C. filispica* showed no difference (Table 1), which is consistent with previous researches (Hao et al., 2020; Ma et al., 2010; Zhou et al., 2012), indicating that compared with the entire community, *C. filispica* had relatively higher tolerance toward high‐intensity trampling. Research on the Tibetan plateau found that as grazing intensified, grasses and legumes were gradually replaced by sedges (Guo et al., 2019), which basically agrees with our result. Sedges were found to be insensitive to degradation, drought, and nitrogen, which leads to the deduction that they have strong adaptability to changes in resources of the environment (Hao et al., 2020; Zong et al., 2014), while our research shows that they also have certain adaptability to physical disturbance such as trampling.

After mild trampling, the stimulation and microenvironment variation can promote the growth of roots, providing nutrients to the aboveground regeneration and leading to a compensating growth (Jiang et al., 2021; Xu et al., 2014). In our study, the growth of DRs fitted this pattern by having a significant increase in both proportion and density after 50 passes (Table 2), which also indicates that the presence of DRs might provide an advantage in minor disturbed environments, thereby becoming a more common trait. The smaller size of DRs was considered an advantage in absorbing nutrients (Masuda et al., 2020), yet in our study, the size of DRs was larger and the color was lighter as the trampling intensified (Table 2). This could be related to the large amount of newly formed DRs, which are generally larger and brighter than the older ones.

### 4.2 More biomass and larger leaves

Plants tend to invest biomass in organs that help to acquire the most limited resource (Bloom et al., 1985; Poorter et al., 2012). When the grazing intensifies, plants often show a decrease in both size and height (Bernhardt‐Römermann et al., 2011; Li et al., 2012), which, on the one hand, is because of the direct damage caused to leaves and stems, but on the other hand, could also be related to this strategy: When under the stress of overgrazing, plants tend to ignore their height and invest resources belowground to maximize their productivity (Westoby et al., 2002).

A previous study showed that species with DRs tend to have less belowground biomass (Gusewell & Schroth, 2017), that is, they are able to meet their needs with less belowground input and save resources to fulfill the growth of aboveground parts. Similarly, compared with the control group with no trampling, individuals with DRs increased their aboveground biomass after 50 and 200 passes, which could also be explained by that they can still meet their belowground needs under mild disturbance and invest in the aboveground competition. Large carbon costs were required to produce these short‐lived roots with high physiological activity (Funayama‐Noguchi et al., 2015). Therefore, the fact that individuals with DRs did not show a difference in control group could be explained by the extra energy invested belowground, which could be fully used once the disturbance occurred, therefore getting an advantage in the competition: Once trampled, the aboveground biomass of individuals with DRs showed an increase before the final decrease, while those without DRs showed only a downtrend.

In addition to the aboveground biomass increase in sedges with DRs, the length, width, and thickness of leaves also showed a positive correlation with the proportion and number of DRs (Figure 2), which is consistent with the previous findings: During the degradation of alpine grassland, the leaf area and aboveground biomass of sedges remained stable or even increased while the other functional groups showed a significant downward trend (Hao et al., 2020; Ma et al., 2010; Zhou et al., 2012). This indicates that DRs might have better resource utilization under disturbed environments and be able to bring extra advantage to the aboveground competition of sedges.

### 4.3 Higher efficiency of nutrient utilization and slenderer roots

Plants with high N and P content of leaves usually have the potential of a higher photosynthetic rate, growth rate, and therefore stronger competitiveness (Wang & Shangguan, 2011), yet plants with lower N and P content tend to become dominant in a nutrient‐limited environment (Yu et al., 2017). Plant growth is mainly limited by N in grassland ecosystems (Bai et al., 2010; LeBauer & Treseder, 2008), and in our study, the aboveground N of individuals with DRs showed a significant decline as the trampling intensified (Figure 4), along with a significant increase in C:N ratios, which can reflect the efficiency of nutrient utilization (Li et al., 2016; Thompson et al., 1997). This could mean that under intensified disturbance, the presence of DRs can greatly improve the efficiency of nutrient utilization. In a previous study, the aboveground P of individuals with DRs was found to be lower than those without and was explained by the inhibition of DR formation owing to the P accumulation in the shoots (Shane et al., 2005), yet our study showed no such trend: It may be that DRs promoted the uptake of P but had not yet reached the threshold to inhibit their formation.

Plants in cold, high‐altitude environments tend to allocate their biomass to belowground and increase the ratio of fine roots (Freschet et al., 2017; Mokany et al., 2006; Ostonen et al., 2017; Reich, 2014), which increases root surface area and helps the plants to absorb nutrients, to adapt to the disturbed environment with strong wind, low temperature, and low nutrients (Körner & Kèorner, 1999). In this study, individuals with DRs appeared to have higher root length, higher surface area, and lower average diameter (Figure 6), which fits with the characteristics of fine roots, therefore may have more advantages in the competition of harsh environment.

### 4.4 Summary: More fitting traits with a tighter correlation

Sedges were proved to have some more suitable strategies for resource acquisition in high‐altitude areas, such as more slender roots, which was believed to be a compensation for the lack of ability to form mycorrhizae (Li et al., 2007; Ma et al., 2010). Plants achieve fast growth by adjusting leaf traits or root traits to acquire resources: They tend to have large leaves which provide them with more light interception, high root area, and low root diameter which provide them with more nutrient absorption, and having both fitting leaf and root traits leads to an even faster growth rate (Simpson et al., 2020). Moreover, plants with high coordination between leaf and root traits were proved to be more adaptive in limited environments (Du et al., 2019). Obviously, in our study, individuals with DRs have multiple abovementioned resource‐acquisitive traits and fit with the following pattern: They have larger leaves, more biomass, higher efficiency of nutrient utilization, and slenderer roots; on top of that, the leaf traits showed coordination with DR properties (Figure 2), and they have a similar tendency of belowground N and P, root length, surface area, volume, and belowground biomass (Figure 5), which peaks after 200 passes, shows they can still adjust to adapt, yet tenser trampling (500 passes) restricted their growth. Therefore, we made a further hypothesis that dauciform roots are related to a better strategy in moderate disturbed environment, and we cannot rule out the possibility that all these traits found to compensate for mycorrhizae may actually result from the formation of dauciform roots.

Interspecific variation was proved to be the dominant factor of leaf functional traits variation, but there was also a substantial amount of intraspecific trait variability that needed to be considered (Liu et al., 2018b). The intraspecific variation of *Cyperaceae* was found under resource limitation (Ji et al., 2020), and *Cyperaceae* with and without DRs were treated as separate functional groups in a previous study (Zemunik et al., 2015). In an experimental environment, dauciform roots were found to have little cost and benefit, therefore may have no particular advantage or disadvantage (Gusewell & Schroth, 2017). Yet, our study showed that, when disturbed in the natural environment, *C. filispica* with DRs showed an apparent variation in biomass, morphological characters, chemical traits, and root properties, which gained a certain advantage.

In conclusion, this study contributes to our understanding of the impacts of dauciform roots, suggesting that DR presence is related to advantages in multiple traits under disturbed environments, which is consistent with and could explain the advantage of sedges found in degraded alpine meadows. Even though the damage was proved to be stable after 2 weeks, the two‐week gap can only reflect the ability of resistance, instead of resilience; therefore, further research is needed after a period of time.

## AUTHOR CONTRIBUTIONS

**Rong Fan:** Conceptualization (equal); formal analysis (lead); investigation (lead); writing – original draft (lead). **Jinguo Hua:** Investigation (equal). **Yulin Huang:** Investigation (equal). **Jiayi Lin:** Investigation (equal). **Wenli Ji:** Conceptualization (lead); supervision (lead).

## CONFLICT OF INTEREST STATEMENT

This manuscript is approved by all authors for publication with no conflict of interest. The work described was original research that has not been published previously, nor under consideration for publication elsewhere.

## Data Availability

Responses of Carex filispica to trampling in alpine meadows based on functional traits, Dryad, Dataset, https://doi.org/10.5061/dryad.9zw3r22hz.
